# The Functional *TP53* rs1042522 and *MDM4* rs4245739 Genetic Variants Contribute to Non-Hodgkin Lymphoma Risk

**DOI:** 10.1371/journal.pone.0107047

**Published:** 2014-09-09

**Authors:** Chuanbo Fan, Jinyu Wei, Chenglu Yuan, Xin Wang, Chuanwu Jiang, Changchun Zhou, Ming Yang

**Affiliations:** 1 Department of Hematology, Shandong Provincial Hospital of Shandong University, Jinan, Shandong Province, China; 2 Department of Hematology, Qingdao Hiser Medical Center, Qingdao, Shandong Province, China; 3 Department of Radiology, Qingdao Hiser Medical Center, Qingdao, Shandong Province, China; 4 Department of Hematology, Qilu Hospital of Shandong University, Qingdao, Shandong Province, China; 5 College of Life Science and Technology, Beijing University of Chemical Technology, Beijing, China; 6 Clinical Laboratory, Shandong Cancer Hospital, Shandong Academy of Medical Sciences, Jinan, Shandong Province, China; University of Texas MD Anderson Cancer Center, United States of America

## Abstract

As a heterogeneous kind of malignances, Non-Hodgkin lymphoma (NHL) is the most common hematologic cancer worldwide with the significantly increased morbidity in China. Accumulated evidences demonstrated that oncoprotein MDM4 plays a crucial role in the TP53 tumor suppressor signaling pathway. An rs4245739 A>C polymorphism locating in the *MDM4* 3′-untranslated region creates a miR-191 target site and results in allele-specific MDM4 expression. In this study, we examined the association between this polymorphism as well as the *TP53* Arg72Pro (rs1042522 G>C) genetic variant and Non-Hodgkin Lymphoma (NHL) risk in a Chinese Han population. Genotypes were determined in 200 NHL cases and 400 controls. Odds ratios (ORs) and 95% confidence intervals (CIs) were calculated by logistic regression. We found significantly increased NHL risk among carriers of the *TP53* 72Pro allele compared with those with the 72Arg allele (*P* = 0.002 for the Pro/Pro genotype). We also observed a significantly decreased NHL risks among carriers of the *MDM4* rs4245739 C allele compared with those with the A allele in Chinese (*P* = 0.014 for the AC genotype). Stratified analyses revealed the associations between these SNPs and NHL risk are especially noteworthy in young or male individuals. Additionally, the associations are much pronounced in NHL patients with B-cell lymphomas or grade 3 or 4 disease. Our results indicate that the *TP53* Arg72Pro and the *MDM4* rs4245739 polymorphisms contribute to NHL susceptibility and support the hypothesis that genetic variants in the *TP53* pathway genes can act as important modifiers of NHL risk.

## Introduction

As a heterogeneous group of malignances, Non-Hodgkin lymphoma (NHL) is the most common hematologic cancer worldwide with the significantly increased morbidity in China [1], [2]. In 2012, the estimated incidence of NHL in China is 41171 cases. NHL derived from T cells or B cells is named as T-cell lymphomas (TCLs) or B-cell lymphomas (BCLs), respectively. TCLs and BCLs are abnormally differentiated from the precursor lymphocytes in different developmental stages. Immune deficiencies and some environmental factors have been identified to be involved in the pathogenesis of certain types of NHL, including human T-cell leukemia/lymphoma virus type 1, human immunodeficiency virus, Epstein-Barr virus and *Helicobacter pylori* [3]–[7]. Moreover, it has been shown that genetic makeup may also play important part during NHL development [8]–[12].

TP53 is one of most important tumor suppressors in human cells, which is essential in maintaining genomic stability and controlling cell growth as well as apoptosis [12], [13]. As a key regulator of the TP53 tumor suppressor signaling pathway, MDM2 could lead to degradation of TP53 through the ubiquitin-proteasome pathway [14], [15]. MDM4 is a structurally homologous protein of MDM2 and can cooperate with MDM2 to inhibit TP53 activities [16], [17]. After interacting with MDM2 protein through the RING finger domain, MDM4 could repress degradation of MDM2 protein [17], [18]. Transgenetic mice with overexpressed MDM4 showed spontaneous tumorigenesis, demonstrating that *MDM4* is an important oncoprotein *in vivo* [19].

A functional single nucleotide polymorphism (SNP) (rs4245739 A>C) in the 3′-untranslated region (3′-UTR) of *MDM4* has been identified, which creates a target binding site of miR-191 [20]. In ovarian cancer, retinoblastoma and esophageal cancer cells, miR-191 could selectively bind to the *MDM4*-C allele mRNA but not the *MDM4*-A allele mRNA, which resulting in a statistically significant increased expression of *MDM4* mRNA and protein among the *MDM4* rs4245739 A allele carriers [20]–[22]. In addition, ovarian cancer patients with rs4245739 AA genotype who do not express the estrogen receptor had a 4.2-fold [95% confidence interval (CI)  = 1.2–13.5; *P* = 0.02] increased risk of recurrence and 5.5-fold (95% CI = 1.5–20.5; *P* = 0.01) increased risk of tumor-related death compared with cases with AC or CC genotype [20]. Our previous studies also indicated that significantly decreased breast cancer and esophageal cancer risks among carriers of the *MDM4* rs4245739 C allele compared with those with the A allele in Chinese [22], [23]. There is also a functional *TP53* SNP at codon 72 (rs1042522 G>C) resulting in Arg>Pro amino acid substitution. The 72Arg allele seems to induce apoptosis with much faster kinetics compared to the 72Pro allele [24], [25], and the 72Pro variant might be more competent during controlling of cell cycle arrest and DNA repair [26], [27].

Considering the essential role of TP53 and MDM4 during carcinogenesis, we hypothesized that the *TP53* Arg72Pro and *MDM4* rs4245739 genetic polymorphism may be involved in NHL development. To test this, we investigated association between these functional SNPs and NHL risk through a case–control study in a Han Chinese population.

## Materials and Methods

### Study subjects

In the study, there are a total of 200 patients with NHL from Shandong Cancer Hospital, Shandong Academy of Medical Sciences (Jinan, Shandong Province, China) and sex- and age-matched (±5 years) 400 controls. Patients were recruited between June 2009 and January 2014 at Shandong Cancer Hospital. Control subjects were randomly selected from a pool of 4500 individuals from a community cancer-screening program for early detection of cancer conducted in Jinan city during the same time period as the patients were collected. The diagnosis of all patients was histologically confirmed. All subjects were ethnic Han Chinese. This study was approved by the Institutional Review Board of Shandong Cancer Hospital, Shandong Academy of Medical Sciences. At recruitment, written informed consent was obtained from each subject.

### SNP genotyping

PCR-based restriction fragment length polymorphism (RFLP) was used to determine *TP53* Arg72Pro and *MDM4* rs4245739 A>C genotypes as previously reported [22], [23]. A 15% random sample was tested by different person, and the reproducibility was 99.8%. Moreover, a 5% random sample was also detected by Sanger sequencing, and the reproducibility was 100% (Figures S1 and S2).

### Statistical analyses

The differences in demographic variables and genotype distributions of *TP53* Arg72Pro and *MDM4* rs4245739 SNPs between NHL patients and controls were examined via Pearson's χ^2^ test. Associations between *TP53* Arg72Pro genotypes or *MDM4* rs4245739 genotypes and NHL susceptibility were calculated by OR and their 95% CIs using the unconditional logistic regression model. All ORs were adjusted for age and sex, where it was appropriate. We tested the null hypotheses of multiplicative gene-gene or gene-covariate interaction and evaluated departures from multiplicative interaction models by including main effect variables and their product terms in the logistic regression model [28], [29]. A *P* value of less than 0.05 was used as the criterion of statistical significance, and all statistical tests were two-sided. All analyses were performed with SPSS software package (Version 16.0, SPSS Inc., Chicago, IL).

## Results

No statistically significant differences were found between NHL patients and controls for the case-control set in terms of median age and sex distribution (both *P*>0.05), which indicating that the frequency matching was adequate (Table 1). For NHL patients, 50 (25.0%) patients were classified into T-cell lymphoma and 150 (75.0%) were classified into B-cell lymphoma. Among cases with B-cell lymphoma, there were 133 (66.5%) patients with diffuse large B-cell lymphoma, 21 (10.5%) patients with follicular lymphoma, 20 (10.0%) patients with marginal zone lymphoma, 11 (2.3%) patients with chronic lymphocytic leukemia/small lymphocytic lymphoma and the remaining 15 (7.5%) other tumors, respectively (Table 1).

**Table 1:** Distribution of selected characteristics among Non-Hodgkin Lymphoma cases and controls.

Variable	Cases (*n* = 200)	Controls (*n* = 400)	*P* a
	No. (%)	No. (%)	
Age (year) b			0.564
≤50	103(51.5)	196(49.0)	
>50	97(48.5)	204(51.0)	
Sex			0.809
Male	128(64.0)	260(65.0)	
Female	72(36.0)	140(35.0)	
Pathology			
T-cell	50(25.0)		
B-cell	150(75.0)		
DLBCL	133(66.5)		
FL	21(10.5)		
MZL	20(10.0)		
CLL/SLL	11(5.5)		
Others	15(7.5)		
Ann Arbor stage			
1+2	84(42.0)		
3+4	116(58.0)		

Note: DLBCL: diffuse large B-cell lymphoma, FL: follicular lymphoma, MZL: marginal zone lymphoma, CLL: chronic lymphocytic leukemia, SLL: small lymphocytic lymphoma.

a Two-sided χ^2^ test.

b Median age of cases is 50 years.

The allelic and genotype frequencies of *TP53* Arg72Pro and *MDM4* rs4245739 A>C SNPs were showed in Table 2. For the *TP53* Arg72Pro polymorphism, the *TP53* 72Pro allele frequency was 0.383 among healthy controls and 0.483 among NHL patients. The frequency for the *MDM4* rs4245739 C allele was 0.069 among healthy controls and 0.033 among NHL patients. All observed genotype frequencies in both controls and cases conform to Hardy-Weinberg equilibrium. We then compared distributions of these *TP53* and *MDM4* genotypes among NHL cases and controls. The frequencies of *TP53* Arg/Arg, Arg/Pro and Pro/Pro genotypes among NHL patients were significantly different from those among controls (χ^2^ = 11.29, *P* = 0.004, *df* = 2). The frequencies of *MDM4* rs4245739 AA, AC and CC genotypes among NHL patients were also significantly different from those among controls (χ^2^ = 6.76, *P* = 0.034, *df* = 2).

**Table 2:** Associations between the *TP53* rs1042522 Arg72Pro and *MDM4* rs4245739 A>C genetic polymorphisms and Non-Hodgkin Lymphoma risk.

Genotype	Cases (*n* = 200)	Controls (*n* = 400)	OR^a^ (95% CI)	*P*
	No. (%)	No. (%)		
*TP53* rs1042522 Arg72Pro				
Arg/Arg	52(26.0)	157(39.3)	1.00 (Reference)	
Arg/Pro	103(51.5)	180(45.0)	1.73(1.16–2.57)	0.007
Pro/Pro	45(22.5)	63(15.7)	2.18(1.32–3.59)	0.002
Pro allele frequency	0.483	0.383		
*MDM4* rs4245739 A>C				
AA	187(93.5)	346(86.5)	1.00 (Reference)	
AC	13(6.5)	53(13.2)	0.45(0.24–0.85)	0.014
CC	0(0)	1(0.3)	NC	NC
C allele frequency	0.033	0.069		

Note: NHL: Non-Hodgkin Lymphoma, OR: odds ratio, 95%CI: 95% confidence interval, NC: not calculated.

a Data were calculated by logistic regression, adjusted for sex and age.

Associations between genotypes of *TP53* Arg72Pro and *MDM4* rs4245739 A>C SNPs and NHL risk were then calculated (Table 2). A significantly increased risk of developing NHL was associated with the *TP53* Arg/Pro genotype (OR = 1.73, 95% CI = 1.16–2.57, *P* = 0.007) or the Pro/Pro genotype (OR = 2.18, 95% CI = 1.32–3.59, *P* = 0.002) compared with the *TP53* Arg/Arg genotype. The *MDM4* rs4245739 C allele was showed to be a protective allele. Individuals having the rs4245739 AC genotype had an OR of 0.45 (95% CI = 0.24–0.85, *P* = 0.014) for developing NHL compared with individual having the rs4245739 AA genotype (Table 2). All ORs were adjusted for age and sex. We also examined whether there are gene-gene interaction between *MDM4* and *TP53* polymorphisms, but the results were negative (*P*_interaction_ = 0.681).

The risk of NHL associated with the *TP53* Arg72Pro or *MDM4* rs4245739 genotypes was further examined by stratifying for age, sex, pathology and Ann Arbor stage (Table 3 and 4). In stratified analyses with age, the *TP53* Arg/Pro and Pro/Pro or the *MDM4* AC and CC genotypes were significantly associated with NHL risk in subjects aged 50 years or younger (*TP53*: OR = 2.46, 95% CI = 1.45–4.16, *P* = 0.001; *MDM4*: OR = 0.42, 95% CI = 0.18–0.99, *P* = 0.048), but not in subjects aged older than 50 years (*TP53*: OR = 1.36, 95% CI = 0.80–2.32, *P* = 0.263; *MDM4*: OR = 0.48, 95% CI = 0.19–1.21, *P* = 0.121). No significant gene-age interaction was observed for both SNPs (*P*_interaction_ = 0.122 or 0.854). Compared with the *TP53* Arg/Arg genotype, a significantly increased risk of NHL was associated with *TP53* Arg/Pro and Pro/Pro genotypes both among males (OR = 1.72, 95% CI = 1.08–2.73, *P* = 0.023), and among females (OR = 2.16, 95% CI = 1.13–4.10, *P* = 0.019). However, compared with the *MDM4* AA genotype, a significantly decreased risk of NHL was associated with the *MDM4* AC and CC genotypes only among males (OR = 0.21, 95% CI = 0.08–0.54, *P* = 0.001), but not among females (OR = 1.35, 95% CI = 0.52–3.47, *P* = 0.536). There was a significant gene-sex interaction for *MDM4* genotypes (*P*_interaction_ = 0.007), but not for *TP53* genotypes (*P*_interaction_ = 0.530).

**Table 3:** Association between *TP53* rs1042522 Arg72Pro variant and NHL risk stratified by selected variables.

Variable	*TP53* Arg72Pro				*P*_interaction_ c
	Arg/Arg^a^	Arg/Pro+ Pro/Pro^a^	OR^b^ (95% CI)	*P*	
Age (year)					0.122
≤50	26/89	77/107	2.46(1.45–4.16)	0.001	
>50	26/68	71/136	1.36(0.80–2.32)	0.263	
Sex					0.530
Male	35/101	93/159	1.72(1.08–2.73)	0.023	
Female	17/56	55/84	2.16(1.13–4.10)	0.019	
Pathology					NC
T-cell	16/157	34/243	2.50(0.78–2.90)	0.226	
B-cell	36/157	114/243	2.02(1.31–3.10)	0.001	

Note: NHL: Non-Hodgkin Lymphoma, OR: odds ratio, 95%CI: 95% confidence interval, NC: not calculated.

a Number of case patients with genotype/number of control subjects with genotype.

b Data were calculated by logistic regression, adjusted for sex and age, where it was appropriate.

c *P* values for gene-environment interaction were calculated using the multiplicative interaction term in SPSS software.

**Table 4:** Association between *MDM4* rs4245739 A>C variant and NHL risk stratified by selected variables.

Variable	*MDM4* rs4245739 A>C				*P*_interaction_ c
	AA^a^	AC+CC^a^	OR^b^ (95% CI)	*P*	
Age (year)					0.854
≤50	96/167	7/29	0.42(0.18–0.99)	0.048	
>50	91/179	6/25	0.48(0.19–1.21)	0.121	
Sex					0.007
Male	123/218	5/42	0.21(0.08–0.54)	0.001	
Female	64/128	8/12	1.35(0.52–3.47)	0.536	
Pathology					NC
T-cell	45/346	5/54	0.77(0.28–2.10)	0.606	
B-cell	142/346	8/54	0.34(0.16–0.74)	0.006	

Note: NHL: Non-Hodgkin Lymphoma, OR: odds ratio, 95%CI: 95% confidence interval, NC: not calculated.

a Number of case patients with genotype/number of control subjects with genotype.

b Data were calculated by logistic regression, adjusted for sex and age, where it was appropriate.

c *P* values for gene-environment interaction were calculated using the multiplicative interaction term in SPSS software.

In the pathology-stratified or grade-stratified analyses, significantly elevated NHL risk was found in the *TP53* Arg/Pro and Pro/Pro genotypes carriers only in BCL cases (OR = 2.02, 95% CI = 1.31–3.10, *P* = 0.001) and cases with grade 3 or 4 disease (OR = 2.46, 95% CI = 1.50–4.05, *P*<0.001) (Table 3). Similar results were also observed for the *MDM4* AC and CC genotypes (Table 4).

## Discussion

In the current study, we investigated the association between *TP53* and *MDM4* functional SNPs and NHL risk in a case-control design. We found significantly increased NHL risk among carriers of the *TP53* 72Pro allele compared with those with the 72Arg allele. Also, we observed a significantly decreased NHL risk among carriers of the *MDM4* rs4245739 C allele compared with those with the A allele in Chinese. These results are in line with functional relevance of *TP53* Arg72Pro as well as *MDM4* rs4245739 polymorphism [20]–[27].

There are several studies which have investigated association between the *TP53* Arg72Pro SNP and NHL susceptibility. However, the results are inconsistent among different ethnic populations [30]–[32]. In Korean, Kim et al examined the association between this *TP53* Arg72Pro polymorphism and NHL risk through a Korean large-scale case-control study (945 cases and 1700 controls) [30]. They found that the *TP53* 72Pro/Pro genotype was associated with increased risk of NHL (*P* = 0.04), which is consistent to our observations in Han Chinese. However, no such association between this polymorphism and NHL risk was found in European Caucasians [31], [32]. The controversial results might be due to differences of ethnic background. Additionally, Weng Y et al evaluate the role of *TP53* Arg72Pro polymorphism in development of hematological cancer through a meta-analysis [33]. They found that significantly increased non-Hodgkin lymphomas risk was found in *TP53* Arg72Pro polymorphism heterozygote model (Arg/Pro vs. Arg/Arg: OR = 1.18, 95% CI = 1.02–1.35) and dominant model (Arg/Pro+Pro/Pro vs. Arg/Arg: OR = 1.18, 95% CI = 1.03–1.34). These results are in line with our findings, indicating that *TP53* Arg72Pro polymorphism may contribute to NHL susceptibility.

Wynendaele et al found that the rs4245739 genetic variant in the *MDM4* 3′UTR creates a miR-191 target site, which was associated with survival of Caucasian ovarian cancer patients [20]. Previously, we also found that this SNP contributes to risk of esophageal squamous cell carcinoma and breast cancer in Chinese populations [22], [23]. These data are consistent with results of the current study and provide evidences supporting that genetic variants located in miRNA target sites may function as a new class of regulators modifying cancer risk.

We expected that there should be a gene-gene interaction between *MDM4* rs4245739 and *TP53* Arg72Pro genetic variants since the functional *TP53* codon 72 Arg>Pro change could depress the activities of TP53 in inducing apoptosis and suppressing transformation [24], [25] and MDM4 can negatively regulate TP53 tumor suppression function [16], [17]. However, we did not observe this interaction, which might be largely due to the relatively small sample size of the current study. Therefore, the findings of our case-control study warrant to be validated in a population-based prospective study in the future.

Considering the gene-gender interaction of the *MDM4* polymorphism, we did find marginal interaction in our previous study on esophageal cancer (*P*_interaction_ = 0.080) [22]. However, in the current study, we only observed significant association between *MDM4* rs4245739 SNP and NHL in males. The most possible explanation might be the relative small sample size of the current study. One hypothesis to explain this is that miR-191 is an estrogen-responsive miRNA [34]. Therefore, there might be much higher expression of miR-191 in female patients compared to male individuals. The high level of miR-191 in cells may greatly repress MDM4 expression and compromise its allele-differential regulation of MDM4. However, experimental evidences are still needed to support this hypothesis.

In summary, our data demonstrated that functional *TP53* Arg72Pro and *MDM4* rs4245739 polymorphisms were significantly associated with NHL risk in a Chinese population. The associations between SNPs and NHL risk are especially noteworthy in young or male individuals. Additionally, the associations are much pronounced in NHL patients with BCL or grade 3 or 4 disease.

## Supporting Information

Figure S1 **Genotyping of the** ***TP53*** **Arg72Pro (rs1042522 G>C) genetic variant.** Up panel, PCR-RFLP results. Low panel, DNA sequencing results.
(PPTX)

Figure S2 **Genotyping of the** ***MDM4*** **rs4245739 A>C genetic variant.** Up panel, PCR-RFLP results. Low panel, DNA sequencing results.
(PPTX)
