# Supplementary figures and images for: The Functional TP53 rs1042522 and MDM4 rs4245739 Genetic Variants Contribute to Non-Hodgkin Lymphoma Risk
Source: PLoS One. 2014 Sep 9;9(9):e107047. doi: 10.1371/journal.pone.0107047 (PMC4159297; doi:10.1371/journal.pone.0107047)

## Slide 1
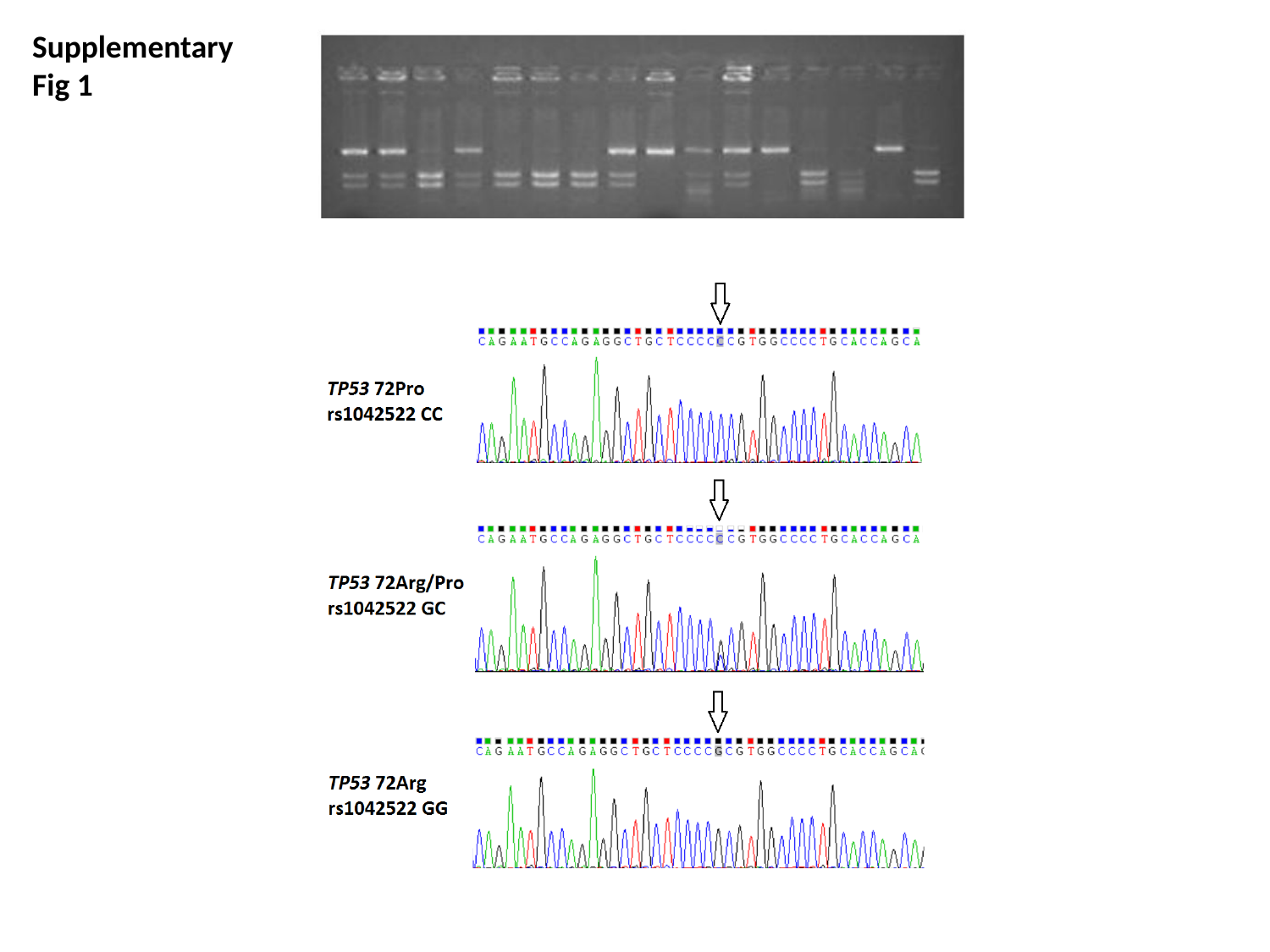

Supplementary Fig 1

Supplement: Figure S1 — Genotyping of the TP53 Arg72Pro (rs1042522 G>C) genetic variant. Up panel, PCR-RFLP results. Low panel, DNA sequencing results. (PPTX) [file pone.0107047.s001.pptx]

## Slide 1
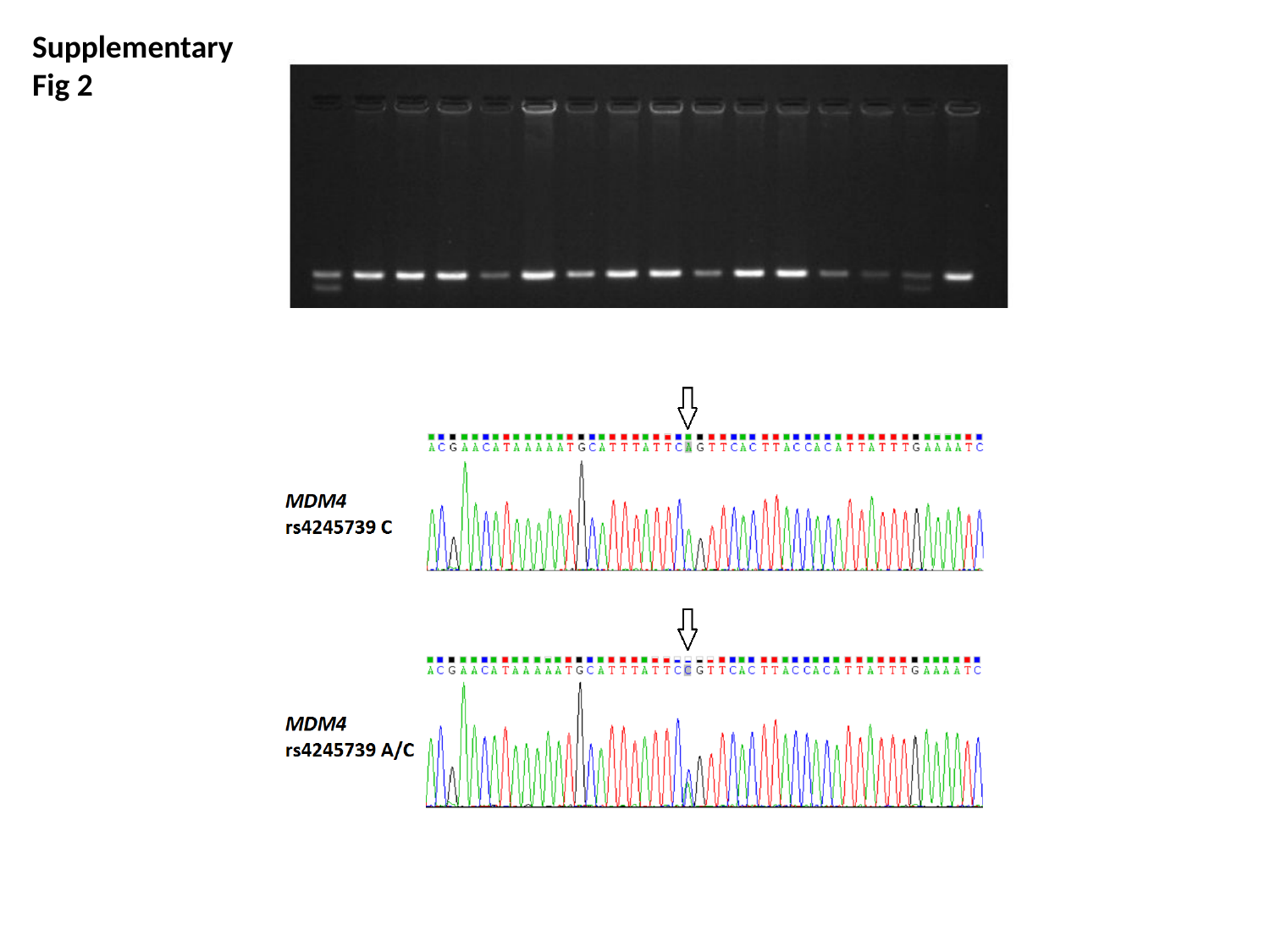

Supplementary Fig 2

Supplement: Figure S2 — Genotyping of the MDM4 rs4245739 A>C genetic variant. Up panel, PCR-RFLP results. Low panel, DNA sequencing results. (PPTX) [file pone.0107047.s002.pptx]
